# Cyclic GMP–AMP Synthase (cGAS) Deletion Reduces Severity in Bilateral Nephrectomy Mice through Changes in Neutrophil Extracellular Traps and Mitochondrial Respiration

**DOI:** 10.3390/biomedicines11041208

**Published:** 2023-04-18

**Authors:** Nattavong Suksawad, Kanyarat Udompornpitak, Natchapon Thawinpipat, Pichaya Korwattanamongkol, Peerapat Visitchanakun, Pornpimol Phuengmaung, Wilasinee Saisorn, Patipark Kueanjinda, Asada Leelahavanichkul

**Affiliations:** 1 Center of Excellence on Translational Research in Inflammation and Immunology (CETRII), Department of Microbiology, Chulalongkorn University, Bangkok 10330, Thailand; 2 Nephrology Unit, Department of Medicine, Faculty of Medicine, Chulalongkorn University, Bangkok 10330, Thailand

**Keywords:** leaky gut, bilateral nephrectomy, cGAS, systemic inflammation, neutrophil

## Abstract

Uremia-induced systemic inflammation is partly caused by the dissemination of microbial molecules such as lipopolysaccharide and bacterial double-stranded DNA from leaked gut damaged by immune cells in response to the microbial molecules. Cyclic GMP–AMP synthase (cGAS) can recognize fragmented DNA and induce cGAMP synthesis for the activation of the stimulator of interferon genes (STING) pathway. To study the effect of cGAS in uremia-induced systemic inflammation, we performed bilateral nephrectomy (BNx) in wild-type and cGAS knock-out mice and found that the gut leakage and blood uremia from both groups were similar. However, serum cytokines (TNF-α and IL-6) and neutrophil extracellular traps (NETs) decreased significantly in cGAS^−/−^ neutrophils after stimulation with LPS or bacterial cell-free DNA. Transcriptomic analysis of LPS-stimulated cGAS^−/−^ neutrophils also confirmed the down-regulation of neutrophil effector functions. The extracellular flux analysis showed that cGAS^−/−^ neutrophils exhibited a higher respiratory rate than wild-type neutrophils despite having similar mitochondrial abundance and function. Our results suggest that cGAS may control effector functions and the mitochondrial respiration of neutrophils in response to LPS or bacterial DNA.

## 1. Introduction

Acute and chronic kidney injury are common health-care problems worldwide that induce uremia, the accumulation of uremic toxins such as urea and creatinine in the blood caused by renal failure, and lead to severe clinical manifestations [1]. Uremic complications such as protein-energy wasting, anorexia, anemia, vascular calcification, atherosclerosis, cardiovascular diseases, and endocrine disorders are reportedly associated with systemic inflammation. There are several mechanisms that cause uremia-induced systemic inflammation in kidney diseases [2,3,4], but one emerging cause is the translocation of pathogenic molecules such as bacterial lipopolysaccharide (LPS) or fungal β-glucan from the leaky gut that has been damaged by the immune cell-mediated attack of enterocytes [5]. Uremic toxins have been shown to damage enterocytes, disrupting permeability and causing holes in the gut barrier that allow pathogenic molecules, including endotoxin and bacterial cell-free DNA, to enter the bloodstream and cause systemic inflammation [6,7,8,9,10]. Furthermore, whole viable bacteria were found in the blood of bacteremia patients and a uremia-induced gut leakage mouse model [11]. These studies have elucidated that systemic inflammation following kidney injury is possibly caused by uremic toxins which can damage the gut barrier and allow pathogenic molecules to leak into the bloodstream and activate immune cells.

When activated by pathogenic molecules in the bloodstream during uremia, innate immune cells—particularly macrophages and neutrophils—can undergo degranulation and phagocytosis, respectively, promoting systemic inflammation. In neutrophils, the formation of neutrophil extracellular traps (NETs) or NETosis is defined as a self-sacrifice mechanism in which chromatin along with antibacterial proteins are released to catch and destroy pathogens. NETosis is evidently mediated by reactive oxygen species (ROS), NADPH oxidase (NOX), neutrophil elastase (NE), and myeloperoxidase (MPO) [12,13,14,15]. Failure to clear NETosis, on the other hand, can initiate an immune response against damage-associated molecular patterns (DAMPs), such as fragmented DNA, histones, and other intracellular proteins [16], potentially leading to the development of autoimmune diseases, diabetes, atherosclerosis, and vasculitis [17]. During uremia, DAMPs from dead cells and pathogen-associated molecular patterns (PAMPs) from microbes have been shown to activate neutrophils and NETs formation [18,19,20,21], possibly via MPO and NE induction [22]. However, the precise mechanisms by which uremia directly or indirectly affects NETosis in response to systemic infection remain unclear.

One of the most extensively studied danger signal sensors in recent years is cyclic GMP–AMP (cGAMP) synthase (cGAS), which recognizes fragmented DNA specifically and induces cGAMP synthesis as a secondary messenger [23,24] for the activation of stimulator of interferon genes (STING), resulting in type I interferon (IFN-I) production [25]. In a mouse model of pulmonary lung infection, cGAS was demonstrated to be an essential facilitator of inflammation induced by PAMPs, such as Pseudomonas aeruginosa-derived DNA. Nonetheless, DAMPs from stressed or apoptotic cells, such as nuclear or mitochondrial DNA (mtDNA), have been shown to induce IFN-I production in a cGAS-dependent manner [26,27]. In a cecal ligation and puncture (CLP)-induced sepsis mouse model, cGAS recognized mtDNA in stressed lung epithelial cells and facilitated lung inflammation via the STING pathway [28]. These studies definitively demonstrate that cGAS can recognize DNA from both self and non-self sources and, as a result, activate cellular responses.

Recently, innate immune cell dysfunctions have been shown to link with the uremic toxins and microbes leaked from the damaged gut of patients undergoing prolonged hemodialysis [29]. Our recently published investigation on the effect of bacterial cell-free DNA in different systemic inflammation models strongly supports this postulation. For example, in the ischemia-reperfusion (IR) kidney injury mouse model, we found that serum dsDNA induced NETosis in Syk- and NF-κB-dependent manners [21]. In addition, we reported that a dextran sulfate solution (DSS)-induced gut leakage, in combination with gut microbiome dysbiosis, and caused a release of bacterial cell-free DNA and uremic toxins into the bloodstream, thereby increasing uremia severity in macrophage- and TLR9-dependent manners [8]. Despite this, the molecular basis for the induction of inflammation and uremic-induced systemic inflammation remains unclear. To address this issue, the present study investigated the role of neutrophils in the BNx-induced gut leakage-associated systemic inflammation mouse model and cultured neutrophils exposed to pathogenic molecules, such as LPS and bacterial cell-free DNA, to mimic the components of bacterial gut leakage. Our results suggest that cGAS may mediate NETosis and negatively regulate mitochondrial respiration, resulting in less severity in BNx mice. Furthermore, cGAS may be a potential therapeutic target for preventing the progression of BNx-mediated systemic inflammation.

## 2. Materials and Methods

### 2.1. Animals and Animal Model

Male 8-week-old C57BL/6 mice were used in this study with permission from the Institutional Animal Care and Use Committee of the Faculty of Medicine, Chulalongkorn University, Bangkok, Thailand (CU-ACUP No. 018/2564; approval date: 30 September 2021) and in compliance with the animal care and use protocol of the United States National Institutes of Health (NIH). Wild-type C57BL/6J mice were purchased from Nomura Siam (Bangkok, Thailand) and cGAS knockout (cGAS^−/−^) mice with a C57BL/6J background were kindly provided by Paludan (Aarhus University, Aarhus, Denmark). Bilateral nephrectomy (BNx) or a sham operation (renal identification before the abdominal suture) was performed on the mice, followed by a post-operative subcutaneous injection of fentanyl (0.03 mg/kg of body weight) in 0.5 mL normal saline solution (NSS) as previously described [11,30,31,32]. At 48 h after surgery, the mice were sacrificed while under isoflurane anesthesia for sample collection.

### 2.2. Mouse Sample Analysis

Renal injury was assessed by measurement of serum creatinine and blood urea nitrogen levels using QuantiChrom assays (DICR-500 and DIUT-500, respectively, Bioassay, Hayward, CA, USA). Serum cytokines were measured by an enzyme-linked immunosorbent assay (ELISA) (Invitrogen, Carlsbad, CA, USA). Liver damage was evaluated by an EnzyChrom alanine transaminase assay (EALT-100, Bioassay, Hayward, CA, USA). Gut leakage was determined from (1) the detection of a nonabsorbable high-molecular-weight fluorescein isothiocyanate-dextran (FITC-dextran) in serum after an oral administration, (2) LPS, (3) bacteremia, and (4) the bacterial cell-free DNA in the blood serum, as previously described [11]. For FITC-dextran assays, 0.5 mL of FITC-dextran (4.4 kDa; 46944, Sigma-Aldrich, St. Louis, MO, USA) at 25 mg/mL was orally administered 3 h prior to blood collection. The detection of FITC-dextran was performed under a fluorescence spectroscope (Varioskan Flash, Thermo Scientific, Waltham, MA, USA) using excitation and emission wavelengths of 485 and 528 nm, respectively. A standard curve based on known concentrations of FITC-dextran was also generated for quantification. Serum LPS was determined using a HEK-Blue LPS detection kit (InvivoGen, San Diego, CA, USA). Bacterial burdens were evaluated by incubating 25 mL of blood in blood agar for 24 h at 37 °C before colony enumeration. To determine bacterial cell-free DNA, serum DNA was extracted with 5 M potassium acetate/acetic acid buffer and quantified using a Nanodrop 100 spectrophotometer (Thermo Scientific, Waltham, MA, USA). Then, the presence of bacterial cell-free DNA was detected using a 16S rRNA primer pair (forward: 5′-GATGAACGCTGGCGGCGTGC-3′, reverse: 5′-CAATCATTTGTCCCACCTTC-3′) in a quantitative real-time polymerase chain reaction (qRT-PCR) using the QuantStudio 6 Flex Real-time PCR System, as previously described [8]. The standard curve derived from known values of bacterial cell-free DNA was also generated for quantification. Briefly, the standard curve was constructed by the QuantStudio™ Design & Analysis Software v1.4.3 (Thermo Fisher Scientific, Waltham, MA, USA) using serial dilutions of standard bacterial cell-free DNA derived from *Escherichia coli* (ATCC 25922; Manassas, VA, USA) which were incubated in tryptic soy broth (Oxoid, Basingstoke, Hampshire, UK) overnight.

Neutrophils were isolated from blood samples using Polymorph-prep (Alere Technologies AS, Oslo, Norway) and their morphology was observed, as described previously [21,33,34]. Then, the dsDNAs in the isolated neutrophils were stained with QuantiT™ PicoGreen reagent (Thermo Fisher Scientific, Waltham, MA, USA), whereas the nuclei were stained with 4′,6-diamidino2-phenylindole (DAPI) (Sigma-Aldrich, St. Louis, MO, USA), for assessment of neutrophil extracellular traps (NETs).

### 2.3. In Vitro Experiments on Neutrophils

For neutrophil isolation, 1 mL of 3% Thioglycolate (Sigma-Aldrich, St. Louis, MO, USA) was intraperitoneally injected in mice from both strains, and the peritoneal neutrophils were harvested after 2 h post-injection by lavages of the peritoneal cavity with 20 mL of cold phosphate buffer solution (PBS) after sacrificing with a cardiac puncture under isoflurane anesthesia as described previously [35]. The cells were centrifuged and washed with PBS before being resuspended in Roswell Park Memorial Institute (RPMI)-1640 media containing 25 mM HEPES, L-Glutamine (Hyclone, Marlborough, MA, USA), and 10% fetal bovine serum (FBS) (Gibco Life Technologies, Paisley, UK). In the 24-well plate containing poly-L-lysine-coated cover glass, neutrophils (1 × 10^5^ cell/well) were stimulated with various NETs stimulators, including (1) 50 ng/mL of phorbol myristate acetate (PMA) (Sigma-Aldrich, St. Louis, MO, USA) as a potent NETs inducer (positive control), (2) 500 ng/mL of LPS (*Escherichia coli* 026:B6; Sigma-Aldrich, St. Louis, MO, USA), (3) 5 ng/µL of bacterial DNA, and (4) 500 ng/mL of LPS together with 5 ng/µL of bacterial DNA, and incubated at 37 °C with 5% CO_2_ for 2 h. Cytokines and dsDNA in the supernatant were measured by an ELISA and Picogreen assay kit (Invitrogen, Carlsbad, CA, USA) according to the manufacturer’s protocol. For NETs evaluation, neutrophils were stained with DAPI for nuclei, antibodies against neutrophil elastase (ab68672) and myeloperoxidase (ab25989) together with secondary antibodies, including Alexa Fluor 488 goat anti-rabbit IgG (ab150077) and Alexa Flour 647 goat anti-mouse IgG (ab150115). All DAPI and all antibodies were purchased from Sigma-Aldrich (St. Louis, MO, USA). Cells were visualized and images were recorded under confocal fluorescent microscopy (ZEISS LSM 800; Carl Zeiss, Jena, Germany). The fluorescent intensity of NE and MPO was scored as previously described [36].

### 2.4. Gene Expression and 2′3′- Cyclic Guanosine Monophosphate–Adenosine Monophosphate (cGAMP)

The expression of genes of interest was assessed using a real-time quantitative reverse transcription polymerase chain reaction (qRT–PCR) and primer pairs as previously described [37]. Briefly, total RNA was extracted using Trizol, quantified using NanoDrop ND-1000 (Thermo Fisher Scientific, Waltham, MA, USA), converted into cDNA using reverse transcriptase, and amplified in PCR using an SYBR Green system (Applied Biosystem, Foster City, CA, USA). The quantification of mRNA was based on the ∆∆CT method that uses *β-actin* as a housekeeping gene. The primers used in this study are listed as follows: *Pad4* (forward: 5′-ACAGGTGAAAGCAGCCAGC-3′, reverse: 5′-AGTGATGTAGATCAGGGCTTGG-3′), *Nfκb* (forward: 5′-CTTCCTCAGCCATGGTACCTCT-3′, reverse: 5′-CAAGTCTTCATCAGCATCAAACTG-3′), *Tlr4* (forward: 5′-GGCAGCAGGTGGAATTGTAT-3′, reverse: 5′-AGGCCCCAGAGTTTTGTTCT-3′), cyclic cGAS pair 9 (forward: 5′-ATGTGAAGATTTCYGCTCCTAATGA-3′, reverse: 5′-GAAATGACTCAGCGGATTTCCT-3′), *Prkaa1* (forward: 5′-AGAGGGCCGCAATAAAAGAT-3′, reverse: 5′-TGTTGTACAGGCAGCTGAGG-3′), *Prkaa2* (forward: 5′-TGGCTGCCTTCTTATGCTTT-3′, reverse: 5′-GCTTTGAAACGGCTTCTCAC-3′), and *β-actin* (forward: 5′-GGACTTCGAGCAAGAGATGG-3′, reverse: 5′-AGCACTGTGTTGGCGTACAG-3′). Additionally, 2′3′-cGAMP was determined using an ELISA kit from Cayman Chemical (Ann Arbor, MI, USA).

### 2.5. Mitochondrial Evaluation and Extracellular Flux Analysis

Mitochondrial DNA (mtDNA) extraction was performed using a Tissue Genomic DNA extraction mini kit (Favorgen Biotech, Wembley, WA, Australia), followed by total DNA quantification using NanoDrop ND-100 (Thermo Fisher Scientific, Waltham, MA, USA). The qRT-PCR was performed as described previously [37] using the following primer pairs: mtDNA (forward: 5′-CGTACACCCTCTAACCTAGAGAAGG-3′, reverse: 5′-GGTTTTAAGTCTTACGCAATTTCC-3′) and β2-microglobulin (*β2m*) (forward: 5′-TTCTGGTGCTTGTCTCACTGA-3′, reverse: 5′-CAGTATGTTCGGCTTCCCATTC-3′). The amount of mtDNA was calculated using the ∆∆CT method that uses *β2m* for normalization, as previously described [38]. Additionally, the mitochondrial membrane potential was analyzed using MitoTracker Red as previously described [37]. Briefly, 200 nM of Mitotracker Red CMxROS (Molecular Probes, Inc., Eugene, OR, USA) was used to stain mitochondria, followed by incubation at 37 °C for 15 min before cell fixation using cold methanol at −20 °C. Mitochondria were measured by a microplate reader at an excitation wavelength of OD_579_ nm.

To explore the impact of LPS and bacteria-free DNA on neutrophil energy status, the Seahorse XFp Analyzer (Agilent, Santa Clara, CA, USA) was used in extracellular flux analysis to determine mitochondrial activity and glycolysis via the oxygen consumption rate (OCR) and extracellular acidification rate (ECAR), respectively, as described previously [39,40,41,42]. Due to the short life span of neutrophils, LPS (*E. coli* 026:B6) (Sigma-Aldrich, St. Louis, MO, USA) at 500 ng/mL with or without bacterial DNA at 5 ng/µL were added to the neutrophils (1 × 10^4^ cells/well) right before incubation with the standard reagents of Seahorse XF analysis and Seahorse substrates (glucose, pyruvate, and L-glutamine) (Agilent, 103575–100) under pH 7.4 at 37 °C for 1 h prior to the challenge with different metabolic compounds, including oligomycin, carbonyl cyanide-4-(trifluoromethoxy)-phenylhydrazone (FCCP), and rotenone/antimycin A for mitochondrial reactions, and glucose, oligomycin, and 2-Deoxy-d-glucose (2-DG) for glycolysis intervention. The concentration of metabolic compounds used was determined according to the manufacturer’s instructions. Data analysis was conducted using Seahorse Wave 2.6 software based on the following equations: maximal respiration = (OCR between FCCP and rotenone/antimycin A)—(OCR after rotenone/antimycin A); respiratory reserve = (OCR between FCCP and rotenone/antimycin A)—(OCR before oligomycin); and glycolysis capacity = ECAR between oligomycin and 2-DG. Glycolytic and mitochondrial ATP production rates were calculated as described in the Seahorse manufacturer’s instructions.

### 2.6. RNA Sequencing and Functional Analysis

An RNA sequencing analysis was performed to determine the influence of LPS on the neutrophils of cGAS^−/−^ mice. Briefly, total RNA was extracted from cGAS^−/−^ neutrophils stimulated with or without 100 ng/mL of LPS (*E. coli* 026: B6, Sigma-Aldrich, St. Louis, MO, USA) for 2 h using an RNeasy mini kit (Qiagen), followed by RNA library preparation and sequencing using Illumina NextSeq 500 performed at Omics Sciences and Bioinformatics Center, Chulalongkorn University, Bangkok, Thailand. The mRNA analysis was conducted based on triplicate neutrophil samples. The sequencing quality was determined using FastQC, and the raw sequencing reads were mapped and aligned against the Mus musculus reference genome GRCm39 using STAR [43], followed by gene quantification against the reference mouse transcriptome using Kallisto [44]. Read counts were normalized and analyzed for differentially expressed genes (DEGs) using the edgeR package [45] and limma-voom package [46,47]. Genes were considered significantly (*p* value < 0.01) differentially expressed when the log2 fold change was less than − 2 or greater than 2, representing down- or up-regulation, respectively. The clustering of DEGs was performed using Euclidean distance and the Ward.D2 method in the ComplexHeatmap package [48]. Then, up- and down-regulated DEGs from neutrophil clusters were used for functional analysis against the Gene Ontology database [49,50] using the enrichr package [51,52,53]. All analyses were carried out using the R program version 4.0.4 unless otherwise specified.

### 2.7. Statistical Analysis

The mean ± standard error of the mean (SEM) was used in the data presentation unless otherwise specified. The statistical significance of group differences was determined using a one-way analysis of variance (ANOVA), followed by Tukey’s analysis for comparison of multiple groups. The Student’s *t*-test or Wilcoxon’s test was used to compare the two groups, as appropriate. Statistical analyses were carried out using SPSS 11.5 software (SPSS, Chicago, IL, USA), Graph Pad Prism version 7.0 software (La Jolla, CA, USA), or stat package in R program, as appropriate. A *p* value less than 0.05 was considered statistically significant. Reports of statistical analysis performed in the figures are available in Supplementary Materials (Tables S1 and S2).

## 3. Results

### 3.1. After Bilateral Nephrectomy, cGAS Deficient Mice Had Similar Leaky Gut Severity but Less Prominent Systemic Inflammation and NETosis than Wild-Type Mice

**Figure 1:**
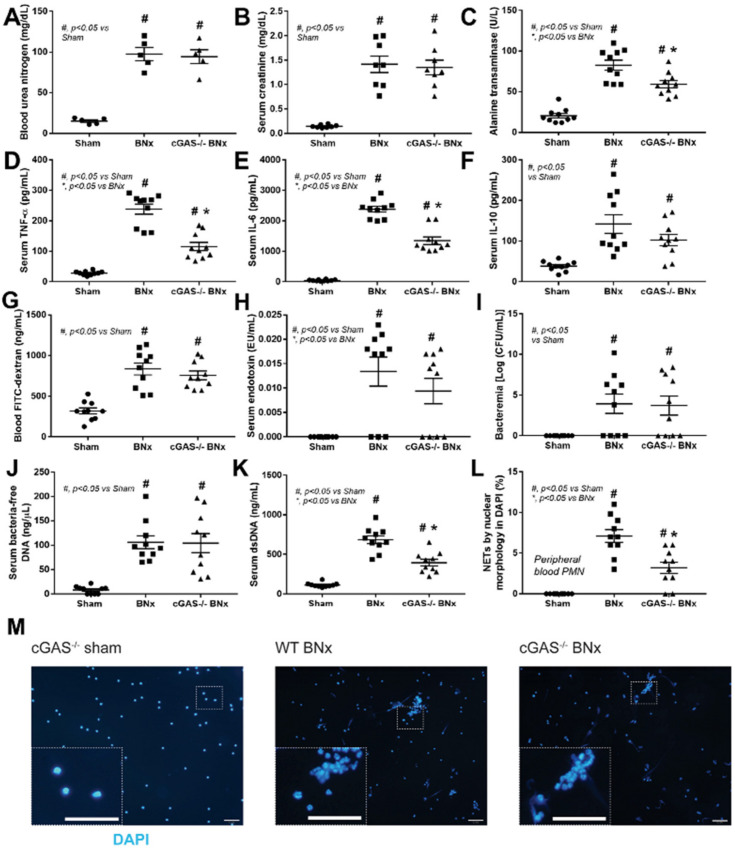
Characteristics of mice after bilateral nephrectomy. Bilateral nephrectomy (BNx) was performed (BNx) on wild-type (WT) and cGAS deficient (cGAS^−/−^) mice. cGAS^−/−^ mice were the sham control (sham). After 48 h of BNx or sham, the mice were sacrificed and biological samples were collected to determine uremia (blood urea nitrogen and serum creatinine) (**A**,**B**), liver damage (serum alanine transaminase) (**C**), serum cytokines (TNF-α, IL-6, and IL-10) (**D**–**F**), and gut barrier defect (FITC-dextran assay, endotoxemia, bacteremia, and bacteria-free DNA) (**G**–**J**). The parameters of neutrophil extracellular traps (NETs), including serum dsDNA (**K**) and NETs (**L**,**M**). (**L**) Indicates the number of cells stained with DAPI for nuclear morphology observation (n = 10 cells/group); (**M**) shows the representative images of those used in (**L**) (original magnification 200×; bar indicates 50 μm in length). The sham data of WT mice are not shown due to their non-difference to cGAS^−/−^ sham mice. *, *p* < 0.05 vs. BNx group; #, *p* < 0.05 vs. sham group. The statistical significance of the difference between the groups was determined by a one-way ANOVA with Tukey’s analysis.

At 48 h after BNx, cGAS^−/−^ and WT mice developed similar severity in uremia, as indicated by an increase in blood urea nitrogen and creatinine (Figure 1A,B, respectively), but had significantly lower alanine transaminase levels, indicating less liver damage (Figure 1C). This was supported by significantly lower levels of serum pro-inflammatory cytokines such as TNF-α and IL-6 (Figure 1D,E, respectively), but not IL-10 (Figure 1F). BNx also induced gut leakage, as evident by elevated levels of blood FITC-dextran, endotoxin, bacteremia, and bacterial cell-free DNA (Figure 1G–J) in both WT and cGAS^−/−^ mice. Similar to the pro-inflammatory cytokines, serum dsDNA was significantly lower in cGAS^−/−^ mice than in WT mice (Figure 1M), which led us to further examine NETosis. Observation of the morphology of peripheral blood neutrophils revealed that NETs formation was significantly lower in neutrophils from cGAS^−/−^ mice than in WT mice (Figure 1L–M). These results suggest that cGAS may play a role in the NET formation in the neutrophils from the BNx-induced acute kidney injury model.

### 3.2. cGAS^−/−^ Neutrophils Exhibited Less LPS-Induced NETs Formation than in WT Cells Because of No Synergy between LPS and Bacterial DNA

**Figure 2:**
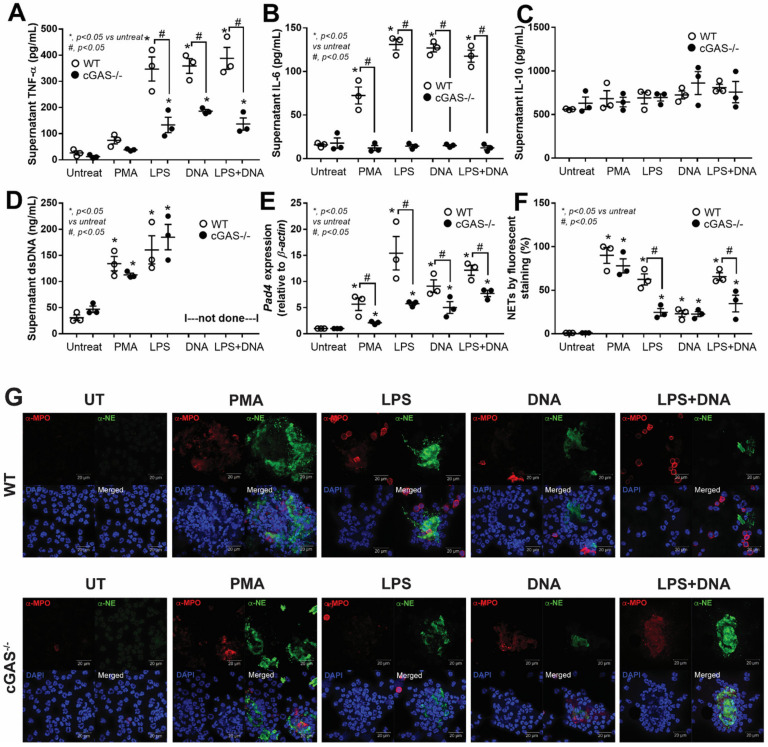
Characteristics of neutrophils under BNx-like conditions. Neutrophils isolated by peritoneal lavage from wild-type (WT) and cGAS deficient (cGAS^−/−^) mice were incubated for 2 h with media control (Untreated or UT), phorbol myristate acetate (PMA; an activator for neutrophil extracellular traps), lipopolysaccharide (LPS), bacterial cell-free DNA (DNA), or LPS with DNA (LPS + DNA). Supernatant cytokines (TNF-α, IL-6, and IL-10) (**A**–**C**) and NET parameters, including supernatant dsDNA (**D**), peptidyl arginine deiminase 4 (*Pad4*) expression (**E**), and NETs (**F**), which were estimated from the fluorescent staining of anti-myeloperoxidase (α-MPO) and anti-neutrophil elastase (α-NE). The representative fluorescent images of NETs in neutrophils (original magnification 630×; bar indicates 20 μm) are shown (**G**). The nucleus is in blue, α-MPO is in red, and α-NE elastase is in green. *, *p* < 0.05 vs. control group (Untreated); #, *p* < 0.05 vs. WT group. The statistical significance of the difference between groups was determined by a one-way ANOVA with Tukey’s analysis.

Because NET formation was more prominent in WT mice over cGAS^−/−^ mice after BNx, we hypothesized that cGAS might regulate NETs in neutrophils and conducted an investigation in vitro. Peritoneal neutrophils were collected from WT and cGAS^−/−^ mice and stimulated in vitro by a potent NETs inducer, PMA, as a positive control, followed by an assessment of supernatant cytokines and NETosis. We found that supernatant TNF-α and IL-10 levels were similar in both WT and cGAS^−/−^ neutrophils (Figure 2A,C), except for lower IL-6 levels in cGAS^−/−^ neutrophils (Figure 2B). In terms of NETosis, both WT and cGAS^−/−^ neutrophils had comparable levels of dsDNA and NETs formation (Figure 2D,F), with the exception of the lower *Pad4* gene in cGAS^−/−^ neutrophils (Figure 2E). In addition to the *Pad4* gene, fluorescent staining with anti-MPO and anti-NE antibodies was used to assess MPO and NE, two essential proteins involved in NETosis, in PMA-activated neutrophils, with MPO and NE levels showing virtually similar levels in both WT and cGAS^−/−^ mice (Figure 2G). When neutrophils from cGAS^−/−^ mice were stimulated with LPS, bacterial DNA, or LPS + DNA, they produced less TNF-α and IL-6 cytokines but not IL-10 (Figure 2A–C). For NETosis in both WT and cGAS^−/−^ neutrophils, the dsDNA appeared to be unaffected by LPS stimulation (Figure 2D). On the other hand, cGAS^−/−^ neutrophils expressed significantly less *Pad4* in all three different treatment conditions (Figure 2E), as did NET formation (Figure 2F), with the exception of bacterial DNA stimulation. The MPO and NE levels were also significantly lower in LPS- or LPS + DNA-stimulated cGAS^−/−^ neutrophils than in WT ones (Figure 2G).

### 3.3. cGAS^−/−^ Neutrophils Responded to LPS Stimulation despite Lower Nfκb Expression

**Figure 3:**
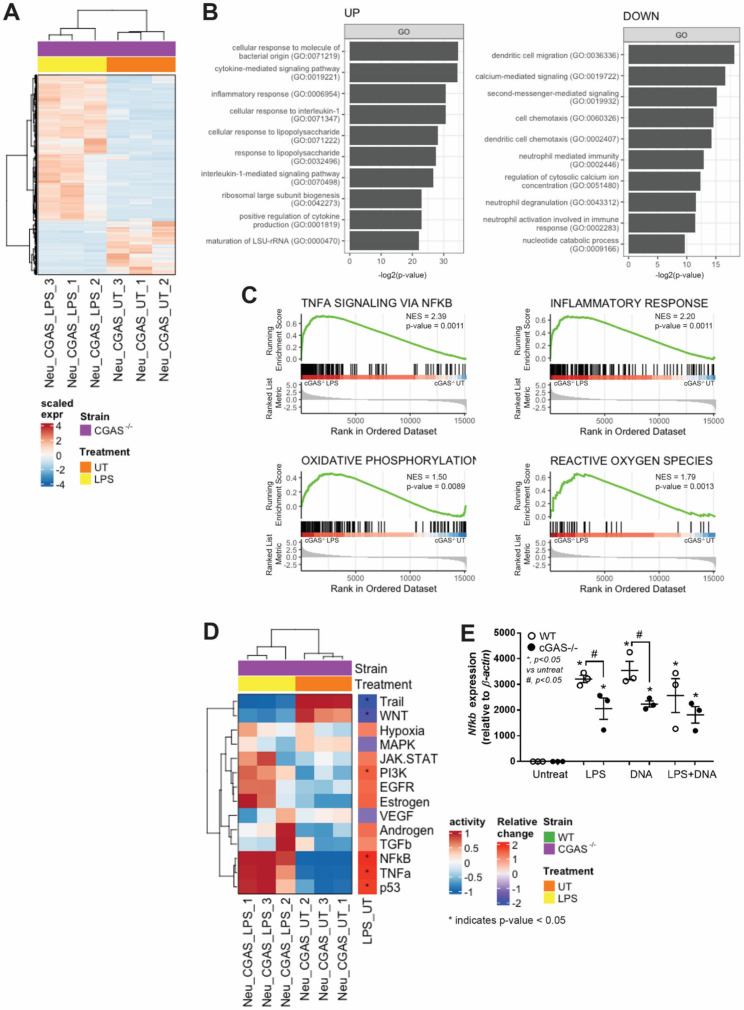
Transcriptome profiles of LPS-activated cGAS^−/−^ neutrophils. Peritoneal neutrophils from cGAS^−/−^ mice were treated with media control (UT) or lipopolysaccharide (LPS) for 2 h followed by RNA sequencing and pathway analysis. Differentially expressed genes (746 up-regulated genes and 271 down-regulated genes) are shown in a heatmap (**A**) The over-representation analysis (ORA) of up-and down-regulated genes from cGAS^−/−^ neutrophils treated with LPS in (**A**) are shown, presenting the top 10 most significant (*p* value < 0.05) biological processes based on the Gene Ontology database (**B**). The enrichment plots of selected pathways (with *p* value < 0.05 and FDR-adjusted *p* value < 0.05) from gene set enrichment analysis (GSEA) using the fold-change of genes from cGAS^−/−^ neutrophils (LPS vs. UT) (**C**). The predicted pathway activity as determined by PROGENy based on RNA-seq data (**D**). The Student’s *t*-test was used to calculate the statistical significance of the difference between activity scores from the LPS vs. UT groups. * indicates *p* value < 0.05. The expression levels of *Nfκb* in neutrophils after incubation with media control (Untreated), LPS (LPS), bacterial cell-free DNA (DNA), or LPS + DNA (**E**). *, *p* < 0.05 vs. control group (Untreated); #, *p* < 0.05 vs. WT group. The statistical significance of the difference between the groups is determined by a one-way ANOVA with Tukey’s analysis.

To investigate the underlying molecular mechanism of cGAS^−/−^ neutrophils in response to LPS, we employed transcriptome analysis based on high-throughput RNA sequencing of the total RNA samples from cGAS^−/−^ neutrophils stimulated with or without LPS for 2 h. According to DEG analysis followed by unsupervised clustering, we found up-regulated (n = 746) and down-regulated (n = 271) genes that were differentially expressed (Figure 3A). Gene Ontology (GO) over-representation analysis (ORA) was then performed on the two groups of DEGs to identify their biological functions in cGAS^−/−^ neutrophils in response to LPS stimulation. The results showed that, when cGAS^−/−^ neutrophils were stimulated with LPS, they increased the expression of the genes involved in the cellular response to bacterial molecules (GO:0071219), particularly to LPS (GO:0071222 and GO:0032496) (Figure 3B, left panel). This result was consistent with our previous finding that the TNF-α level increased in response to LPS stimulation even in neutrophils with cGAS deletion (Figure 3A). In addition, the up-regulated genes are also involved in the interleukin (IL)-1-mediated signaling pathway (GO:0070498) and in response to IL-1 (GO:0071347). In contrast, the analysis of down-regulated genes from LPS-stimulated cGAS^−/−^ neutrophils revealed that the neutrophils had less neutrophil activation activities, such as neutrophil-mediated immunity (GO:0002446), neutrophil degranulation (GO:0043312), and neutrophil activation involved in immune response (GO:0002283) (Figure 3B, right panel). These results supported our recent finding reporting that NET formation was reduced after LPS stimulation in cGAS^−/−^ neutrophils (Figure 2F). Intriguingly, genes involved in calcium signaling (GO:0019722 and GO:0051480) and secondary messenger-mediated signaling (GO:0019932) were down-regulated. We also performed gene set enrichment analysis (GSEA) [54] against the hallmark gene sets collected from the mouse molecular signatures database (MSigDB) [55]. The results revealed that TNF-α signaling via NF-κB and inflammatory response was ranked at the topmost enriched gene sets (Figure 3C, top panel), consistent with our previous GO-ORA and pro-inflammatory cytokine results. Notably, two mitochondrial activity-related pathways, oxidative phosphorylation, and reactive oxygen species, were also found to be enriched (Figure 3C, bottom panel), suggesting the involvement of mitochondria in response to LPS stimulation in cGAS^−/−^ neutrophils.

In addition to GO-ORA, we employed PROGENy [56] to predict the pathway activity of cGAS^−/−^ neutrophils in response to LPS. The analysis revealed that NF-κB pathway activity increased significantly in cGAS^−/−^ neutrophils followed by TNF-α, p53, and, to a lesser extent, PI3K pathways after LPS stimulation; conversely, Trail and WNT pathway activity decreased significantly (Figure 3D). The expression levels of *Nfκb* in cGAS^−/−^ neutrophils increased significantly after LPS stimulation (Figure 3E), confirming our pathway activity prediction results. However, the *Nfκb* expression level, as it partially pertains to the activation of the NF-κB signaling pathway known to be located downstream of NETosis, LPS-TLR4, and cGAS-STING pathway activation, increased significantly in WT neutrophils but decreased significantly in neutrophils with cGAS deletion following LPS, bacterial DNA, or LPS + DNA stimulation (Figure 3E). Our findings suggest that cGAS has a regulatory role in neutrophils, as it can partially regulate cellular activation through the NF-κB signaling pathway. In summary, we demonstrated that bacterial DNA does not promote NETosis synergistically in the presence of LPS. According to our transcriptome analyses, in the absence of cGAS in neutrophils, LPS stimulation still induces pro-inflammatory cytokine production, oxidative phosphorylation, and reactive oxygen species pathways but reduces neutrophil effector functions, which may be mediated in part through the NF-κB signaling pathway.

### 3.4. LPS or Bacterial Cell-Free DNA Activated NET Formation Partly through TLR4-Mediated cGAMP That Induced Mitochondrial Injury and Reduced Mitochondrial Activity

**Figure 4:**
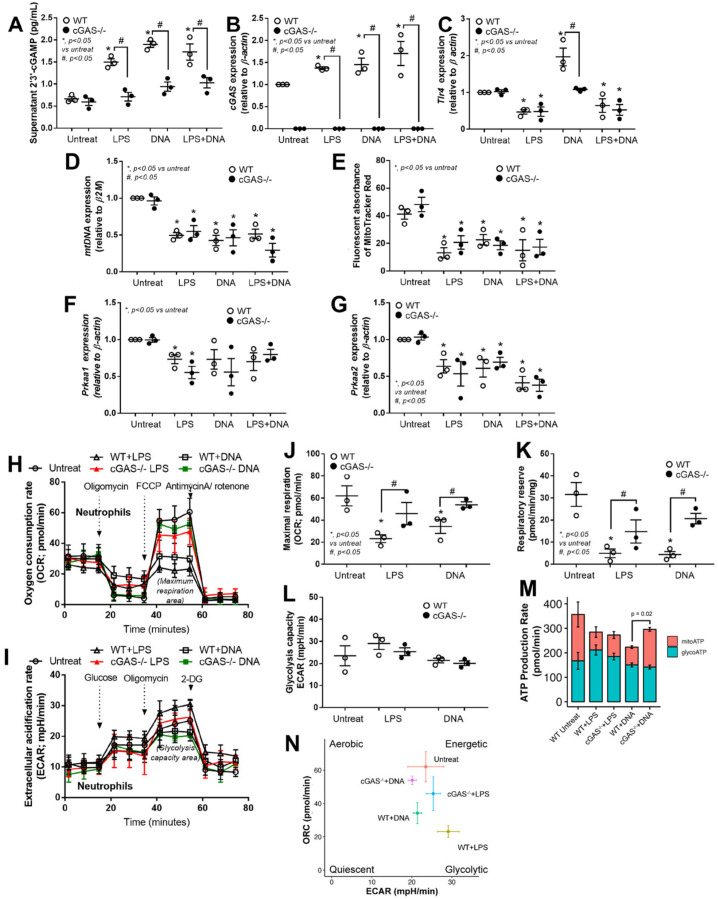
cGAS attenuated mitochondrial respiration but not glucose metabolism in neutrophils. Peritoneal neutrophils from wild-type (WT) and cGAS deficient (cGAS^−/−^) mice were incubated with media control (Untreated), lipopolysaccharide (LPS), bacterial cell-free DNA (DNA), or LPS with DNA (LPS + DNA) and measured for supernatant 2′3′-cyclic GMP-AMP (2′3′-cGAMP) (**A**), expression of cyclic GMP-AMP synthase (cGAS) (**B**), Toll-like receptor 4 (*Tlr4*) (**C**), mitochondrial DNA (mtDNA) (**D**), mitochondrial transmembrane potential (fluorescent MitoTracker red) (**E**), protein kinase AMP-activated catalytic subunit alpha 1 and alpha 2 (*Prkaa1* and *Prkaa2*, respectively) (**F**,**G**), and extracellular flux analyses, including oxygen consumption rate (OCR) (**H**), extracellular acidification rate (ECAR) (**I**), maximal respiration (**J**), respiratory reserve (**K**), and glycolysis (**L**). *, *p* < 0.05 vs. control group (Untreated); #, *p* < 0.05 vs. WT group. The statistical significance of the difference between the groups is determined by a one-way ANOVA with Tukey’s analysis. (**M**) The total rate of ATP production was calculated as the sum of the glycolytic ATP rate (glycoATP) formation and mitochondrial-derived ATP rate (mitoATP) formation that was estimated from the ATP-linked OCR, assuming a P/O ratio of 2.75. Error bars are SEM (n = 3 biological replicates). *p* value of the Student’s *t*-test (mitoATP; WT + DNA vs. cGAS^−/−^ + DNA). (**N**) Bioenergetic profiles of the neutrophils in each condition. Data shown are the mean ± SEM (n = 3 biological replicates).

Because we showed that cGAS ablation hampered NETosis and that the secondary messenger-mediated signaling was reduced according to the transcriptome analysis, we, therefore, measured the production of 2′3′-cGAMP, a secondary molecule synthesized by cGAS that induces the STING pathway and inflammation. When WT neutrophils were stimulated with LPS, bacterial DNA, or both, the 2′3′-cGAMP synthesis increased significantly (Figure 4A); however, the synthesis was stopped nearly completely in neutrophils with cGAS deletion even in the presence of stimuli (Figure 4A,B). Moreover, we also examined the expression level of *Tlr4*, a cognate receptor of LPS to better understand the upstream mechanism of NETs formation. Indeed, the Tlr4 expression in neutrophils from WT and cGAS^−/−^ mice was suppressed after stimulation with LPS alone or LPS + DNA; bacterial DNA stimulation, on the other hand, highly induced *Tlr4* expression in WT neutrophils. Ligand-induced *Tlr4* expression was diminished in cGAS^−/−^ neutrophils (Figure 4C), indicating that cGAS may exert a regulatory role on *Tlr4* expression in activated neutrophils. Moreover, there is no synergistic effect on *Tlr4* expression in neutrophils when stimulated with both LPS and bacterial DNA.

### 3.5. cGAS Attenuated Mitochondrial Respiration but Not Glucose Metabolism in Neutrophils

Mitochondria are a source of cellular energy, but they can be damaged and release mtDNA and other molecules as DAMPs when under stress or under apoptotic conditions. Our transcriptomic analysis confirmed that LPS stimulation induced two mitochondria-related pathways—oxidative phosphorylation and reactive oxygen species—in cGAS^−/−^ neutrophils (Figure 3C). Although we did not analyze the transcriptome of WT neutrophils, we included both WT and cGAS^−/−^ neutrophils in subsequent experiments to investigate the mitochondrial number and functions. Following stimulation with LPS, DNA, or both, the mitochondria in these neutrophils decreased significantly, as evidenced by a reduction in the levels of mitochondrial DNA (Figure 4D). In addition, the transmembrane potential also significantly decreased after neutrophils became active (Figure 4E). Despite these changes, however, there was no significant difference in the levels of mitochondrial DNA or membrane potential between WT and cGAS^−/−^ neutrophils.

The loss of mitochondrial membrane potential signals stress in mitochondrial biogenesis in which AMP-activated protein kinase (AMPK) is served as a metabolic sensor in response to changes in the AMP/ATP ratio [57]. AMPK is a heterotrimeric holoenzyme consisting of catalytic alpha subunits and regulatory beta and gamma subunits [58]. Skin fibroblasts from a patient with a mutation in the alpha catalytic subunits encoded by *PRKAA1* and *PRKAA2* were reported to exhibit a lower oxygen consumption rate and mitochondrial respiratory chain activity, especially the activity of complex I + complex III [59], implying that *PRKAA1* and *PRKAA2* can be used as surrogate markers to monitor AMPK activity and mitochondrial respiration. To indirectly evaluate mitochondrial respiration, we assessed the expression levels of *Prkaa1* and *Prkaa2*. The expression levels of *Prkaa1* significantly decreased in both WT and cGAS^−/−^ neutrophils stimulated with LPS but not DNA or LPS + DNA (Figure 4F). The expression levels of *Prkaa2*, on the other hand, significantly decreased in both WT and cGAS^−/−^ neutrophils stimulated with all ligands (Figure 4G). Again, no difference in the expression levels of these genes was observed between WT and cGAS^−/−^ neutrophils after stimulation.

To examine direct mitochondrial respiration and energy metabolism, we performed a mitochondrial efflux experiment on WT and cGAS^−/−^ neutrophils under different treatment conditions. The results showed that the maximal respiration and respiratory reserve significantly decreased in ligand-stimulated WT neutrophils but they were recovered in the absence of cGAS in neutrophils (Figure 4H,J,K). On the other hand, the glycolysis activity was not affected by ligand stimulation and cGAS deletion (Figure 4I,L). Then, we estimated glycolytic and mitochondrial ATP production rates from our neutrophils’ metabolic data and found that DNA-stimulated cGAS^−/−^ neutrophils significantly increased their mitochondrial ATP production rate compared to DNA-stimulated WT cells (Figure 4M). Finally, the bioenergetic profiles of the neutrophils in each group clearly indicated shifts in the bioenergetic phenotype of neutrophils where activated cGAS^−/−^ neutrophils changed their bioenergetic profiles from the glycolytic phenotype toward energetic phenotype (Figure 4N). Taking these results together, cGAS may negatively control oxygen consumption and mitochondrial ATP production in neutrophils in response to stimulation with bacterial cell-free DNA.

## 4. Discussion

In this study, we reported that uremia and leaky gut were found significantly in both WT and cGAS^−/−^ mice undergoing bilateral nephrectomy (BNx) when compared to the sham. Uremia-induced leaky gut can be partially initiated by uremic toxins, such as serum urea, nitrogen, and serum creatinine, that are excreted unintentionally into the intestine during renal impairment [29,60]. Interestingly, these toxins have been demonstrated to cause damage to enterocytes [61,62,63] and to selectively allow the growth of some bacteria with detoxification properties [11]. Consequently, the openings of enterocytes and translocation of pathogen molecules from the gut can occur, leading to uremia and systemic inflammation. Based on our findings and other reports, BNx may initiate kidney injury, the elevation of blood uremic toxins, gut damage and leakage, uremia, and systemic inflammation in our mouse model.

Uremia induced systemic inflammation in our BNx mouse model, as demonstrated by elevated serum cytokines, gut leakage, and bacteremia as well as dsDNA and NET-forming cells. Interestingly, a significant reduction in serum TNF-α, IL-6, dsDNA, and NET-forming cells in blood cells was observed in cGAS^−/−^ mice. Among various potent NETs inducers, LPS and cell-free DNA (from host or bacteria) have been extensively studied [64,65] and identified in blood during uremia [66,67]. Uremia-induced NETosis can be initiated by (a) the host’s necrotic renal parenchymal cells containing histone and self-nucleic acids from an injured kidney [68], (b) pathogen molecules from a damaged/leaky gut [21], and/or (c) direct activation by various uremic toxins [69]. Thus, a significant reduction in serum dsDNA in cCGAS^−/−^ mice may contribute in part to the lower NET formation of blood cells in systemically inflamed BNx mice.

Additionally, the observation of lower NET-forming cells, IL-6, and TNF-α cytokines in the blood serum from BNx cGAS^−/−^ mice prompted us to examine neutrophils. The ex vivo stimulation of neutrophils from WT and cGAS^−/−^ mice showed that NET formation, IL-6, and TNF-α production were impaired in the absence of cGAS. We have previously reported similar findings in macrophages in which the global genetic deletion or pharmacological inhibition of cGAS effectively attenuated TNF-α and IL-6 expression in macrophages [70,71]. In addition, *Pad4* expression was significantly reduced in cGAS^−/−^ neutrophils in our study. The *Pad4* gene has been reported to be dependent on IL-6 in neutrophils extracted from a rheumatoid arthritis mouse model [72], and it is essential for NET formation against bacterial infection in neutrophils [73]. A reduction in *Pad4* expression in cGAS^−/−^ neutrophils suggests that cGAS may be located upstream of the IL-6/PAD4/NET axis.

Despite our limited samples in the transcriptomic experiment, our analysis revealed NF-κB activity and mitochondrial-related activities after LPS stimulation in cGAS^−/−^ neutrophils. However, when compared to activated WT neutrophils, the *Nfκb* expression was significantly lower, suggesting the partial control of cGAS on neutrophil function through *Nfκb*. In our data, *Tlr4* was suppressed in WT and cGAS^−/−^ neutrophils when activated with LPS, which is in line with another report on alveolar macrophages from a rodent model of lung injury after resuscitation from hemorrhagic shock [74]. Several reports have also demonstrated that *Tlr4* expression is controlled by LPS concentration and NF-κB activity [72,75]. Although we found that cGAS highly induced *Tlr4* expression, additional experiments are required to demonstrate a clear link between cGAS and *Tlr4* expression. It has also been reported that the activation of the cGAS-STING pathway induces the TBK1-mediated activation of IRF3 and NF-κB pathways and results in type I interferon and pro-inflammatory cytokine production [73,76]. Combining our findings and reports with others, we propose that cGAS controls NET formation through the STING/TBK1/NF-κB/IL-6/PAD4 axis.

During BNx-induced uremia, both LPS and bacterial cell-free DNA are present in the bloodstream and might have a synergistic effect in inducing NETs formation. Our ex vivo experiments demonstrated that LPS or LPS + DNA, but not bacterial DNA alone, can induce NET formation to the same extent as the positive control PMA. This emphasizes that LPS is a potent NET inducer, which is consistent with other studies [12,76]. Again, our transcriptomic analysis revealed that the genes related to second-messenger-mediated signaling, including secondary molecules such as cGAMP, decreased substantially. We indirectly verified this finding by measuring 2′3′-cGAMP production and found that it was decreased in ligand-activated cGAS^−/−^ neutrophils compared to WT neutrophils. Although bacterial cell-free DNA has been demonstrated to be a potent TLR4 inducer that facilitates NET formation through the activation of TLR4-mediated downstream signaling pathways [21,77], we found that bacterial cell-free DNA induced NET formation to a similar extent in neutrophils from both strains, suggesting no synergistic effect of bacterial cell-free DNA on LPS in NET formation.

Furthermore, we observed a significant reduction in the relative number of mitochondria in neutrophils from both strains following stimulation with different ligands, consistent with the downregulation of genes related to the neutrophil activities identified in our transcriptome analysis. Additionally, we found a decrease in mtDNA levels, potentially attributed to LPS stimulation, which has been previously shown to inhibit mtDNA synthesis in a rat systemic inflammation model [78]. Interestingly, prior research has linked LPS stimulation to decreased CaMK-IV expression, a calcium signaling regulatory molecule, suggesting a potential association between calcium signaling and mtDNA synthesis. Our transcriptomic data revealed a down-regulation of genes related to calcium-mediated signaling in LPS-stimulated cGAS^−/−^ neutrophils, suggesting that LPS stimulation may inhibit the calcium signaling pathway in this model. However, further investigation is required to fully elucidate the role of calcium signaling in cGAS^−/−^ neutrophils.

Intriguingly, our findings showed that cGAS^−/−^ neutrophils exhibit less severe NETosis, a higher oxygen consumption rate, and higher mitochondrial ATP production, despite a similar mitochondrial abundance, mitochondrial transmembrane potential, and *Prkaa1* and *Prkaa2* expression. The cGAS/STING pathway has been shown to associate with both acute and chronic inflammation, as demonstrated in sepsis-induced acute lung injury and fibrosis in several organs [79,80,81]. Furthermore, it has been shown to be correlated with PRKAA, as demonstrated by the impaired cGAS/STING activation in AMPK inhibitors or AMPK deficient cells [82]. In the present study, LPS activation-induced cell injury may have led to a decrease in *Prkaa1* and *Prkaa2* expression, impairing the ability of the cells to switch to an alternative energy source [83]. Although no synergistic effect was observed between LPS and the bacterial cell-free DNA in NET formation, cGAS may be one of the receptors responsible for enhanced neutrophil activity after LPS stimulation. Moreover, our previous report on macrophages showed that cGAS plays a role in energy conservation, as evidenced by reduced glycolysis activity after cGAS deletion [37]. Taken together, the role of cGAS in cellular function may be cell-context dependent.

In addition to mitochondrial respiration, neutrophils can generate energy via alternative metabolic pathways such as (a) glycolysis, which converts glucose into pyruvate and produces ATP as a byproduct, (b) the pentose phosphate pathway, which generates NADPH and ribose-5-phosphate for nucleotide synthesis, (c) the fatty acid oxidation pathway, which breaks down fatty acids to produce ATP, and (d) amino acid catabolism, which involves the breakdown of amino acids to produce intermediates for the tricarboxylic acid cycle and ATP [84]. These pathways are used by neutrophils during cellular differentiation and effector functions. For example, immature neutrophils use fatty acid oxidation-mediated oxidative phosphorylation for energy production [85]. However, during NET formation they become dependent on glycolysis, bypassing the pentose phosphate pathway [86]. In degranulating neutrophils, both glycolysis and mitochondrial ATP production are involved [87]. In this study, cGAS deletion increased the mitochondrial ATP production rate but not the glycolytic ATP production rate in DNA-activated neutrophils. It also shifted activated neutrophils from a glycolytic towards an energetic state, which has been observed with less pro-inflammatory cytokines and NET formation. Our findings imply that cGAS plays a prominent role in neutrophils’ energy metabolism.

## 5. Conclusions

**Figure 5:**
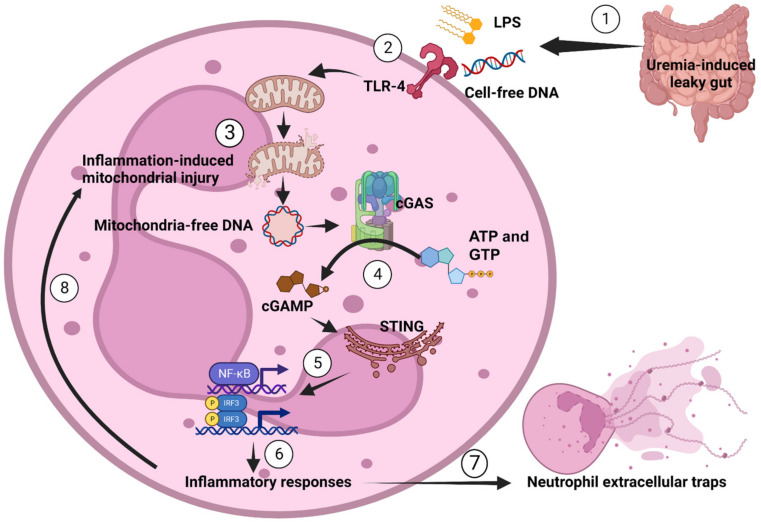
The proposed hypothesis demonstrates a possible impact of cGAS on neutrophils during uremia-induced leaky gut. As the pathogen molecules, including lipopolysaccharide (LPS) and bacterial cell-free DNA (cell-free DNA), from the gut (1) are recognized by TLR4 (2), they cause inflammation-induced mitochondrial injury and the generation of mitochondrial DNA (mtDNA) (3) that is recognized by cGAS (4) and induces cyclic GMP-AMP (cGAMP). Then, cGAMP activates the stimulator of interferon genes (STING), resulting in the transcription of pro-inflammatory genes (5) that further induce inflammation (6), causing neutrophil extracellular traps (NETs) (7) and/or activating more mitochondrial injury (8) and repeating the processes for NETosis (cell death by NETs).

In conclusion, we demonstrated that LPS and bacterial cell-free DNA, which may originate from gut translocation induced by uremia, can increase NETosis in the BNx mouse model. However, the severity of the disease was reduced in mice deficient in cGAS. Deletion of cGAS restored mitochondrial respiration and shifted the bioenergetic phenotype from a glycolytic to an energetic state, which was accompanied by the decreased production of proinflammatory cytokines and NET formation in neutrophils (as shown in Figure 5). These findings highlight the significance of coordinated cellular responses in maintaining cellular homeostasis after an inflammatory insult. They also offer valuable insights into the interplay between neutrophils and mitochondria in the context of systemic inflammation induced by uremia, which is found particularly in patients undergoing frequent hemodialysis [29]. Finally, targeting cGAS may be an attractive strategy for attenuating uremia-induced acute kidney injury.

## Data Availability

Raw RNA sequencing data were generated at the Omics Science and Bioinformatics Center, Chulalongkorn University, Bangkok, Thailand. RNA sequencing data of neutrophils was deposited in the Gene Expression Omnibus database (GSE220693).

## Supplementary Materials

The following supporting information can be downloaded at: https://www.mdpi.com/article/10.3390/biomedicines11041208/s1, Table S1: Detailed statistical data from all figures; Table S2: Detailed statistical data from Figure 4M.
