# Supplementary material for: Cyclic GMP–AMP Synthase (cGAS) Deletion Reduces Severity in Bilateral Nephrectomy Mice through Changes in Neutrophil Extracellular Traps and Mitochondrial Respiration
Source: Biomedicines. 2023 Apr 18;11(4):1208. doi: 10.3390/biomedicines11041208 (PMC10136362; doi:10.3390/biomedicines11041208)
Supplement: Supplementary file 1 [file biomedicines-11-01208-s001.zip › biomedicines-2288631-supplementary.pdf]

Table S1. Detailed statistical data from figures.

AVONA value	F	DF	p-value
Fig 1A	105	2	<0.0001
1B	30.46	2	<0.0001
1C	44.89	2	<0.0001
1D	72.51	2	<0.0001
1E	171.4	2	<0.0001
1F	11.22	2	0.0003
1G	24.1	2	<0.0001
1H	9.096	2	0.001
1I	5.281	2	0.0116
1J	16.4	2	<0.0001
1K	58.2	2	<0.0001
1L	34.77	2	<0.001
Fig 2A	32.96	9	<0.0001
2B	107.6	9	<0.0001
2C	1.318	9	0.2887
2D	14.39	9	0.0001
2E	14.31	9	<0.0001
2F	29.56	9	<0.0001
Fig 3E	15.96	7	<0.0001
Fig 4A	23.7	7	<0.0001
4B	49.14	7	<0.0001
4C	4.32	7	0.0073
4D	12.88	7	<0.0001
4E	6.854	7	0.0007
4F	2.805	7	0.0415
4G	6.669	7	0.0008
4J	4.95	4	0.0184
4K	9.584	4	0.0019
4L	1.87	4	0.1925

Table S2. Detailed statistical data from Figure 4M.

Parameters	group1	group2	t-statistic	DF	P-value	Adjusted p-value
mitoATP	WT Untreat	WT+LPS	2.10167784	2.68924113	0.137	1
mitoATP	WT Untreat	cGAS-/-+LPS	1.9187538	2.29306467	0.179	1
mitoATP	WT Untreat	WT+DNA	2.27219849	2.04773175	0.148	1
mitoATP	WT Untreat	cGAS-/-+DNA	0.68873902	2.08933053	0.559	1
mitoATP	WT+LPS	cGAS-/-+LPS	-0.5836745	3.41469311	0.596	1
mitoATP	WT+LPS	WT+DNA	0.01121327	2.2673589	0.992	1
mitoATP	WT+LPS	cGAS-/-+DNA	-3.5420488	2.49497939	0.051	1
mitoATP	cGAS-/- +LPS	WT+DNA	1.01700699	2.63148456	0.393	1
mitoATP	cGAS-/- +LPS	cGAS-/-+DNA	-4.1690337	3.11106328	0.023	0.46
mitoATP	WT+DNA	cGAS-/-+DNA	-8.5842557	3.66229177	0.001	0.02
glycoATP	WT Untreat	WT+LPS	-1.1097044	3.26648681	0.342	1
glycoATP	WT Untreat	cGAS-/-+LPS	-0.4764028	2.60417074	0.671	1
glycoATP	WT Untreat	WT+DNA	0.46805426	2.21587378	0.682	1
glycoATP	WT Untreat	cGAS-/-+DNA	0.71778624	2.21765739	0.541	1
glycoATP	WT+LPS	cGAS-/-+LPS	1.0915542	3.45911075	0.345	1
glycoATP	WT+LPS	WT+DNA	2.76564417	2.59288411	0.082	1
glycoATP	WT+LPS	cGAS-/-+DNA	3.16405731	2.59758763	0.062	1
glycoATP	cGAS-/- +LPS	WT+DNA	2.17206117	3.24716521	0.111	1
glycoATP	cGAS-/- +LPS	cGAS-/-+DNA	2.73060872	3.25524456	0.066	1
glycoATP	WT+DNA	cGAS-/-+DNA	0.77815492	3.99993149	0.48	1
